# Lipid metabolism involved in progression and drug resistance of breast cancer

**DOI:** 10.1016/j.gendis.2024.101376

**Published:** 2024-07-15

**Authors:** Wenxiang Fu, Aijun Sun, Huijuan Dai

**Affiliations:** a Renji School of Clinical Medicine, Shanghai Jiaotong University, Shanghai 200127, China; b Department of Thyroid and Breast Oncological Surgery, The Affiliated Huaian Hospital of Xuzhou Medical University, Huai'an Second People's Hospital, Huai'an, Jiangsu 223001, China; c Department of Breast Surgery, Renji Hospital, School of Medicine, Shanghai Jiaotong University, Shanghai 200127, China

**Keywords:** Breast cancer, Ceramide, Cholesterol, Drug resistance, Fatty acids, Lipid metabolism, Tumor progression

## Abstract

Breast cancer is the most common malignant tumor threatening women's health. Alteration in lipid metabolism plays an important role in the occurrence and development of many diseases, including breast cancer. The uptake, synthesis, and catabolism of lipids in breast cancer cells are significantly altered, among which the metabolism of fatty acids, cholesterols, sphingolipids, and glycolipids are most significantly changed. The growth, progression, metastasis, and drug resistance of breast cancer cells are tightly correlated with the increased uptake and biosynthesis of fatty acids and cholesterols and the up-regulation of fatty acid oxidation. Cholesterol and its metabolite 27-hydroxycholesterol promote the progression of breast cancer in a variety of ways. The alteration of lipid metabolism could promote the epithelial–mesenchymal transition of breast cancer cells and lead to changes in the tumor immune microenvironment that are conducive to the survival of cancer cells. While the accumulation of ceramide in cancer cells shows an inhibitory effect on breast cancer. This review focuses on lipid metabolism and elaborates on the research progress of the correlation between different lipid metabolism and the growth, progression, and drug resistance of breast cancer.

## Introduction

Breast cancer is the most common malignant tumor in women. According to statistics from the World Health Organization, breast cancer caused 685,000 deaths worldwide in 2020, which constitutes a serious public health problem. Four markers are regularly used in the international molecular classification of breast cancer, namely estrogen receptor (ER), progesterone receptor (PR), human epidermal growth factor receptor 2 (HER2), and Ki-67. Breast cancer with negative expression of ER, PR and HER2 is called triple-negative breast cancer (TNBC), which is characterized by poor differentiation, high invasiveness, propensity for local and distant metastasis, poor prognosis, and high recurrence rate.^1^Despite advances in diagnosis and treatment, a significant number of patients still experience relapse and chemotherapy resistance after initial treatment, resulting in poor prognosis and reduced overall survival.^2^ To find novel therapeutic targets to inhibit the occurrence and development of breast cancer and combat drug resistance, it is necessary to investigate the occurrence, development and drug resistance mechanism of breast cancer (especially TNBC) to enable patients to obtain better clinical outcomes.

An increasing number of studies have shown that alterations in lipid metabolism, such as fatty acid (FA) metabolism,^3^ play a critical role in the occurrence and development of many diseases, especially malignant diseases represented by cancer. Abnormal lipid metabolism can alter membrane composition, gene expression, signaling pathway activity, and downstream cell function, thus directly affecting the occurrence and development of a variety of disease processes.^4^ Therefore, it is important to clarify the lipid metabolism network in cancer cells, interaction of tumor cells, and tumor microenvironment (TME), as well as lipid disorder in therapeutic response. The following sections focus on lipid metabolism, elaborating on the research progress of the correlation of different lipid metabolism with tumor growth and progression, TME, and drug resistance of breast cancer.

## Lipid metabolism interacts with malignant tumors

Lipids are composed of various types of molecules, such as FA, triacylglycerols, cholesterols, phospholipids, and sphingolipids, and are required to maintain various aspects of cellular structure, energy supply, and signal transduction. Lipid metabolism, including catabolic processes to produce energy and anabolic processes to produce different lipids, is the basis for maintaining normal physiological functions of cells. Plasma membrane lipid components (mainly including phospholipids, glycolipids, and cholesterols) compartmental cellular structures and ensure the orderly progress of biological reactions to maintain cell homeostasis. Disturbed lipid metabolism can alter plasma membrane composition and permeability, which are essential for maintaining cellular function. Lipid metabolism generates an array of biomolecules, many of which act as signaling molecules that help regulate multiple signaling pathways that control cell growth, proliferation, differentiation, apoptosis, motility, inflammation, survival, and membrane homeostasis.^3^

Lipid metabolism plays a crucial role in the occurrence and development of cancer. Lipids provide raw materials for cell membrane generation required for cell replication and energy for cell life activities through fatty acid oxidation (FAO), and they can also increase the production of “lipid second messenger” molecules that mediate oncogenic pathways.^5^ Consequently, the promotion of lipid biosynthesis contributes to the proliferation of tumor cells, while the inhibition of lipid biosynthesis can limit cancer cell survival and inhibit tumor growth. Lipid metabolism in cancer cells can be regulated not only by intracellular oncogenic signals, but also by the input of the TME consisting of various cells, cytokines, growth factors, DNA, RNA, and nutrients, including lipids. Conversely, disorders of lipid metabolism can alter the oncogenic signaling pathways of cancer cells and affect the surrounding normal cells by secreting substances including lipids.^6^

## Role of lipid metabolism in breast cancer progression

Numerous studies have shown that the expression of related enzymes and regulatory factors in the process of lipid biosynthesis and catabolism has a significant impact on the proliferation and metastasis of breast cancer cells (BCCs). The following contents introduce the research progress in the metabolism of FA, triglycerides, cholesterols, sphingolipids, and glycolipids in the occurrence and development of breast cancer.

## FA metabolism

FA, an important component of cellular lipid composition, consists of hydrocarbon chains with a carboxyl group at one end and is essential for the structure and function of living cells. FA is divided into saturated FA and unsaturated FA. Unsaturated FA includes monounsaturated FA and polyunsaturated FA.

FA is metabolized to acetyl-CoA and adenosine triphosphate (ATP) synthesis through the FAO in mitochondria.^7^ In addition to being an important energy source for cells, FA maintains homeostasis in cellular biochemical processes such as biofilm formation and membrane fluidity maintenance and serves as a secondary messenger in signaling pathways.^8^ FA in mammalian cells can be obtained through its synthesis and external uptake. Direct cellular uptake of FAs is a key component of metabolic regulation. FAs are mainly absorbed into cells through cluster of differentiation 36 (CD36, FA translocase), plasma membrane FA-binding protein (FABP), and six FA transporters (FATP1–6).^8^ The synthesis pathway of FA involves the action of fatty acid synthase (FASN) and the utilization of acetyl CoA, nicotinamide adenine dinucleotide phosphate (NADPH), and ATP as substrates, while producing palmitate (a 16-carbon fatty acid).^9^ FA synthesis, uptake, and FAO play important roles in the occurrence and progression of breast cancer, which involves changes in many metabolic processes. The following contents focus on the key transporters or enzymes in the FA metabolism process, and introduce the changes and roles of FA metabolism in breast cancer from three aspects of FA uptake, synthesis, and FAO.

CD36, a scavenger receptor, can take up long-chain fatty acids and oxidized low-density lipoproteins and is involved in regulating lipid uptake, immune recognition, inflammation, molecular adhesion, and apoptosis.^10^ Stearoyl-CoA desaturase-1 (SCD1) is an enzyme that catalyzes the conversion of saturated FA to monounsaturated FA. BCCs can uptake exogenous monounsaturated FA via CD36, but BCCs are highly dependent on the activity of SCD1 in the absence of exogenous monounsaturated FA, and inhibition of SCD1 and CD36 produces a significant anti-tumor synergy in breast cancer.^11^ CD36 is a key gene up-regulated in lapatinib-resistant cells. Drug-resistant cells can survive by up-regulating CD36 expression level, and targeting CD36 can restore the sensitivity of lapatinib-resistant cells to HER2 inhibition.^12^ The nuclear receptor Nur77 is a transcription factor encoded by the immediate early gene Nr4a1 (nuclear receptor subfamily 4 group A member 1), Nur77 interacts with the promoters of CD36 and fatty acid binding protein 4 (FABP4) and inhibits their transcription, thereby hindering fatty acid uptake and inhibiting cell proliferation, and BCC proliferation was accelerated in Nur77 knockdown.^13^ Inhibition of FATP4 and CD36 in BCCs and TME leads to dysregulation of FA transport and intracellular FA transport in TME; FATP4 and CD36 also participate in cancer cell apoptosis through other mechanisms such as SGLT2 (sodium–glucose cotransporter 2) cotransporter and AMPK (AMP-activated protein kinase)/mTOR (mechanistic target of rapamycin kinase) pathway.^14^

FATPs are encoded by solute carrier 27 gene family A (SLC27A), overexpression of either FATP increases fatty acid uptake. During FA uptake, FABP reversibly binds to long-chain acyl CoA or free long-chain fatty acids and transports them into the cell.^15^ FA and estrogen stimulate the expression of FATP1/SLC27A1 (solute carrier family 27 member 1) in TNBC, and FATP1/SLC27A1 expression is higher in the more aggressive MDA-MB-231 cell line,^16^ suggesting that active uptake of FAs plays an important role in breast cancer progression.

In BCCMDA-MB-231 exposed to cancer-associated fibroblasts (CAF), FASN activity is decreased, FABP2 and FABP3 transcriptional expression are decreased, while FATP1/SLC27A1 transcriptional expression is increased.^17^ BCCs can increase the lipolysis of adipocytes, adipocytes produce fatty acids, fatty acids enter cancer cells, and then FAO is increased in cancer cells, thus the survival and growth of cancer cells are promoted.^18^ The key molecule in this process is FABP4, and when it is inhibited, metabolic interactions are reduced.^18^ FABP4 is highly expressed in all subtypes of breast tumor tissue compared with normal breast tissue, and high levels of FABP4 are associated with tumor progression in BCCs.^19^ Lipids from peritumoral adipocytes can be transferred into BCCs, and adipocytes interact with cancer cells to utilize stored lipids to promote tumor progression; increased expression of adipose triglyceride lipase (ATGL) and intracellular fatty acid transport protein FABP5 play a key role in this process.^20^ The expression of FABP6 is inhibited in breast cancer tissues.^21^

FASN is the key enzyme in fatty acid synthesis. Although the phenomenon of reduced activity in TNBC has been demonstrated in many studies, its expression level plays an important role in the development of breast cancer.

Nuclear factor Y (NF-Y), a trimeric protein complex, includes three subunits NF-YA, NF-YB, and NF-YC. NF-YA and sterol regulatory element binding protein 1 (SREBP1) jointly regulate the expression of acetyl-CoA carboxylase (ACACA) and FASN, thereby promoting the adipogenesis of TNBC cells and the progression of breast cancer.^22^ In a clinical study of TNBC patients, investigators showed higher breast cancer proliferation activity with increased expression of FASN.^23^ Animal experiments showed that knockdown of Fasn favored the reduction of BCC proliferation and migration,^24^ and genetic or pharmacological inhibition of Fasn reduced HER2^+^ breast tumor growth in the brain.^25^ In *in vitro* cell culture, leucine deprivation reduced the expression of the adipogenic gene FASN, inhibited the proliferation of TNBC cells, and induced apoptosis *in vivo* and *in vitro*, but overexpression of FASN or palmitic acid supplementation blocked the effects of leucine deprivation on cell proliferation and apoptosis.^26^

FAO occurring within mitochondria is important for both the growth and metastasis of cancer cells, including breast cancer,^6^ and FAO is important for maintaining the self-renewal of breast cancer stem cells (BCSC).^27^

Analysis of BCC lines has shown metastatic TNBC maintains high levels of ATP through FAO, and the mitochondrial FAO and carnitine palmitoyltransferase 1 (CPT1) genes are important in cancer cell metastasis by activating tyrosine protein kinase (SRC) through autophosphorylation of Y419.^28^ Up-regulation of CPT1 expression and enhancement of FAO are both important for maintaining the stemness and trastuzumab resistance of BCCs.^29^ The expression of oncogenic transcription factor MYC is increased in TNBC, and TNBC cells with MYC overexpression are FAO-dependent; pharmacological inhibition of FAO can significantly reduce cancer cell energy metabolism and limit tumor growth.^30^

Increased FA synthesis and uptake and enhanced FAO process promote BCC growth and metastasis. This appears to be due to the increased entry of more FAs into the cells and the accelerated FAO process providing BCCs with more ATP, thus promoting BCC progression. Further research is needed to validate this.

## Triglyceride metabolism

Triglycerides are produced by the esterification of three hydroxyl groups of glycerol with three fatty acid molecules. There are few studies specifically focusing on the role of the triglyceride metabolism pathway in breast cancer. The following content briefly introduces the role of two enzymes related to triglyceride metabolism in breast cancer.

LPIN1, an enzyme that displays phospholipid phosphatase activity, regulates the rate-limiting step in the triglyceride and phospholipid synthesis pathways. It was found that the expression of LPIN1 messenger RNA was significantly elevated in breast cancer tissues, and when it was inhibited, BCC migration was reduced, suggesting a promoting effect of LPIN1-regulated lipid synthesis on BCC migration.^31^ SRC, an oncogene, interacts with LPIN1 and promotes breast cancer development.^32^

Down-regulation of retinoic acid receptor reactor 2 (RARRES2) expression is particularly relevant to breast cancer brain metastasis. The low expression of RARRES2 in TNBC cells with brain metastasis potential leads to the increase of glycerophospholipid level and the decrease of triglyceride level by regulating the PTEN (phosphatase and tensin homolog)-mTOR-SREBP1 signal transduction pathway, thereby promoting the survival of BCCs in the unique brain microenvironment.^33^

Elevated levels of diacylglycerol (glycerophospholipids) promote BCC metastasis. In addition to forming biological membranes, glycerophospholipids also serve as components of membrane surfactants and are involved in the recognition of proteins and signal transduction in cell membranes. Further study on the mechanism of glycerophosphatidylcholine promoting BCC migration is of great significance for understanding BC metastasis.

## Cholesterol metabolism

Cholesterol is a derivative of cyclopentane polyhydrophenanthrene. The liver is the major organ for cholesterol biosynthesis, where cholesterol is synthesized from acetyl-CoA and NADPH by a series of enzymatic reactions with 3-hydroxy-3-methylglutaryl-coa (HMG-CoA) reductase as the key enzyme.^34^ Regulation of cholesterol levels involves transcription factors such as SREBPs and liver X receptor (LXR).^35^ Cholesterol is converted into steroid hormones (progesterone, estradiol, testosterone, cortisol, aldosterone, *etc*.) in the body, and the main route is to be converted into bile acids.^35^

Altered cholesterol metabolism is an important part of lipid metabolic reprogramming in cancer cells, which has a significant impact on cancer cell proliferation, invasion, metastasis, and drug resistance.^35^ The following contents focus on the important proteins/enzymes in cholesterol metabolism, and introduce the role of cholesterol metabolism in breast cancer.

SREBP1, a major transcription factor controlling lipid metabolism, promotes breast cancer growth and metastasis both *in vitro* and *in vivo* and is significantly associated with epithelial–mesenchymal transition (EMT) of cancer cells.^36^ The hyperactivated cholesterol biosynthesis in TNBC is closely related to the nuclear receptor retinoid-related orphan receptor gamma t (RORγ). RORγ, an important activator of the whole cholesterol biosynthesis process, binds to cholesterol biosynthesis genes and promotes the recruitment of SREBP2.^37^ RORγ antagonists in combination with statins (cholesterol-lowering effect) show superior synergistic anti-tumor effects in TNBC.^37^ ATP-binding cassette subfamily A member 9 (ABCA9), a cholesterol transporter mainly present in the endoplasmic reticulum, can inhibit BCC proliferation by inhibiting the translocation of SREBP2 from the endoplasmic reticulum to the nucleus and reducing intracellular cholesterol synthesis.^38^ NUCB2 up-regulates SREBP2 and HMG-CoA reductase (key molecules of cholesterol synthesis) by activating the mTORC1 (mechanistic target of rapamycin complex 1) pathway, leading to increased cholesterol synthesis, which contributes to breast cancer migration and metastasis.^39^

The cholesterol metabolizing enzyme NSDHL (NAD(P)-dependent steroid dehydrogenase-like) is a potential metastatic driver in TNBC. NSDHL is highly expressed in breast cancer tissues, and its function depends on its enzymatic activity in cholesterol biosynthesis.^40^ Steroidogenic acute regulatory protein-related lipid transferase 4 (STARD4) is an important cholesterol transporter involved in the regulation mechanism of intracellular cholesterol homeostasis. It acts as an oncogene in breast cancer, and the up-regulation of its expression is associated with the invasiveness of BCCs.^41^

LXR is a ligand-dependent transcription factor and a homeostatic regulator of cholesterol.^42^ 27-hydroxycholesterol (27HC) is the major metabolite of cholesterol and acts as a ligand for ER and LXR.^43^ ER activation by 27HC induces BCC proliferation, and LXR can reduce cellular cholesterol uptake and increase efflux, inducing EMT and subsequent breast cancer metastasis.^44^ In addition, 27HC inhibits the proliferation and cytotoxic effects of T cells by activating the LXR pathway, thereby promoting an immunosuppressive environment that promotes breast cancer metastasis.^45^ LXR ligands confer resistance to chemotherapy in TNBC cells, and LXRα is both necessary and sufficient for resistance in BCCs.^46^

Increased cholesterol synthesis is closely associated with the progression of breast cancer, Cholesterol metabolite 27HC promotes the progression of breast cancer, through multiple pathways, including promoting proliferation and metastasis and remodeling the microenvironment. Currently, the mechanism of 27HC in the progression of breast cancer,is a hot research topic. In-depth studies of the cancer-promoting mechanism of 27HC will provide new insights and strategies for the treatment of breast cancer, especially TNBC.

## Sphingolipid metabolism

Sphingolipids are amphoteric lipids containing a sphingosine backbone. Sphingolipids include sphingolipids, cerebrosides, and gangliosides (GD). Ceramide (Cer) is the precursor of most sphingolipids. Most studies on sphingolipid metabolism and cancer focus on Cer and GD. This section introduces the correlation between Cer metabolism and breast cancer, GD will be introduced in glycolipid metabolism.

Cer is a key lipid in energy metabolic pathways and signaling cascades, regulates key physiological functions of cells, and is a key regulator of apoptosis and proliferation of cancer cells as well as drug resistance of cancer cells. Sphingolipid levels are generally higher in breast cancer, tissues than in normal breast tissues. The levels of sphingolipids such as Cer, sphingdiene-Cer, and sphingolipids are increased in BBCs compared with normal cells.^47^ Sphingomyelin synthetase 2 (SGMS2) is an important regulator of Cer and sphingomyelin balance. Up-regulation of SGMS2 can significantly reduce Cer synthesis, lead to abnormal apoptotic activity, and promote BCC proliferation.^48^ In addition, increased Cer production correlates with decreased resistance proteins in TNBC cells and sensitizes BCC responses to chemotherapy.^49^ It has been clearly shown that short-chain (C6) Cer can significantly enhance the growth inhibition and apoptosis of BCCs induced by docetaxel.^50^ Super-short chain (C2) Cer significantly inhibits the growth and apoptosis of MDA-MB-231 cells by down-regulating mutant p53 expression and up-regulating pro-apoptotic Bad expression, while C2-Cer-induced dominant senes-like phenotype of Rb causes human breast cancer, MCF-7 (wild-type p53) to escape p53-dependent cell death.^51^

Cer exerts negative regulatory effects on the progression of breast cancer,. It suppresses the growth and drug resistance of BCCs, promotes apoptosis, and influences the fate of cancer cells. Inducing apoptosis in BCCs by promoting ceramide synthesis or inducing exogenous Cer uptake may emerge as a novel therapeutic approach for breast cancer, treatment.

## Glycolipid metabolism

Glycolipids are compounds containing one or more monosaccharide residues that bind to the hydrophobic portion of the lipid through the glycosidic bond. Glycolipids mainly include sphingolipids, galactolipids (distinguished from glycosphingolipids by lack of nitrogen), and glycosylphosphatidylinositols. Current research focuses on glycosylated Cer and GD, and the following contents focus on these two glycolipids.

Glycosphingolipids (also called glycosphingolipids) are lipids that contain at least one monosaccharide residue and Cer. Aberrant sphingolipids are enriched in many cancers. The levels of fucosylceramide and Globo-H ceramide (GHCer) are both increased in breast cancer, tissues, but these two kinds of glycosphingolipid effects related to breast cancer, are different, and the abnormalities of fucosylceramide are specific to breast cancer,.^52^ The experiments demonstrated that the addition of GHCer to endothelial cells induced migration, lumen formation, and mobilization of intracellular Ca^2+^ and induced angiogenesis *in vivo*.^53^ BBCs expressing high levels of GHCer exhibited relatively greater tumorigenicity and angiogenesis compared with cells expressing low levels of GHCer.^53^

Cer galactosyltransferase, an enzyme responsible for the synthesis of galactosylceramide (GalCer), is an important indicator of tumor invasiveness and a potential marker for the prognosis evaluation of lung metastasis of breast cancer.^54^ High expression of this enzyme accompanied by GalCer accumulation in breast cancer MDA-MB-231 cells was found to be associated with a higher proliferation index and a lower number of apoptotic cells, and increased resistance to adriamycin-induced apoptosis *in vitro*.^54^ UDP-glucosylceramide glucosyltransferase, a key enzyme in the synthesis of glycosylated sphingolipids, promotes BCC proliferation and adriamycin resistance by activating the PI3K (phosphoinositide 3 kinase)-AKT (protein kinase B) pathway in cells.^55^

Clinical studies have shown that the expression of asialoganglioside (GD2) is associated with poor prognosis of TNBC patients.^56^ GD2 is a marker of BCSC in TNBC, and this phenotype can be induced by metabolic stress caused by nutrient deficiency.^57^ D3S binds to downstream products GD2 and GD3 to maintain the stem cell phenotype in BCSC.^58^ Furthermore, induction of EMT in transformed human breast epithelial cells significantly increased GD2 and GD3S expression in these cells, suggesting a role for EMT in shaping the phenotype of GD2-positive BCSC.^59^ FAK (focal adhesion kinase)-AKT-mTOR signaling pathway is activated in GD2 positive BCSC and is tightly regulated by ST8 alpha-N-acetyl-neuraminide alpha-2,8-sialyltransferase 1 (ST8SIA1).^60^ The growth and metastasis of TNBC cells *in vivo* can be completely blocked by knocking down ST8SIA1.^60^ Tumor necrosis factor (TNF) induces the overexpression of GD3S in BBCs by activating the NF-κB (nuclear factor kappa B) pathway, and the overexpression of GD3S inhibits the invasion potential of human breast cancer MDA-MB-231 cells by down-regulating intercellular adhesion molecule 1 (ICAM-1).^61^^,^^62^

Interestingly, the inhibition of BC progression by Cer contrasts with the promotion of BC progression by glycosylated Cer. The effects of GD on BCs follow the same trend as glycosylated Cer. We believe that the mechanisms of action of sphingolipids and glycolipids (especially Cer and glycosylated Cer) in BCs warrant further investigation.

## Dysregulation of lipid metabolism promotes EMT

It is now generally accepted that EMT is an important cause of distant metastasis in epithelial cancers, including breast cancer. EMT promotes the occurrence, metastasis, drug resistance, and recurrence of BCCs by changing the gene expression profile of BCCs and regulating the expression of genes related to stemness, migration, and drug resistance.^63^ Here, we briefly introduce the role of lipid metabolism in the EMT of breast cancer.

**Figure 1:**
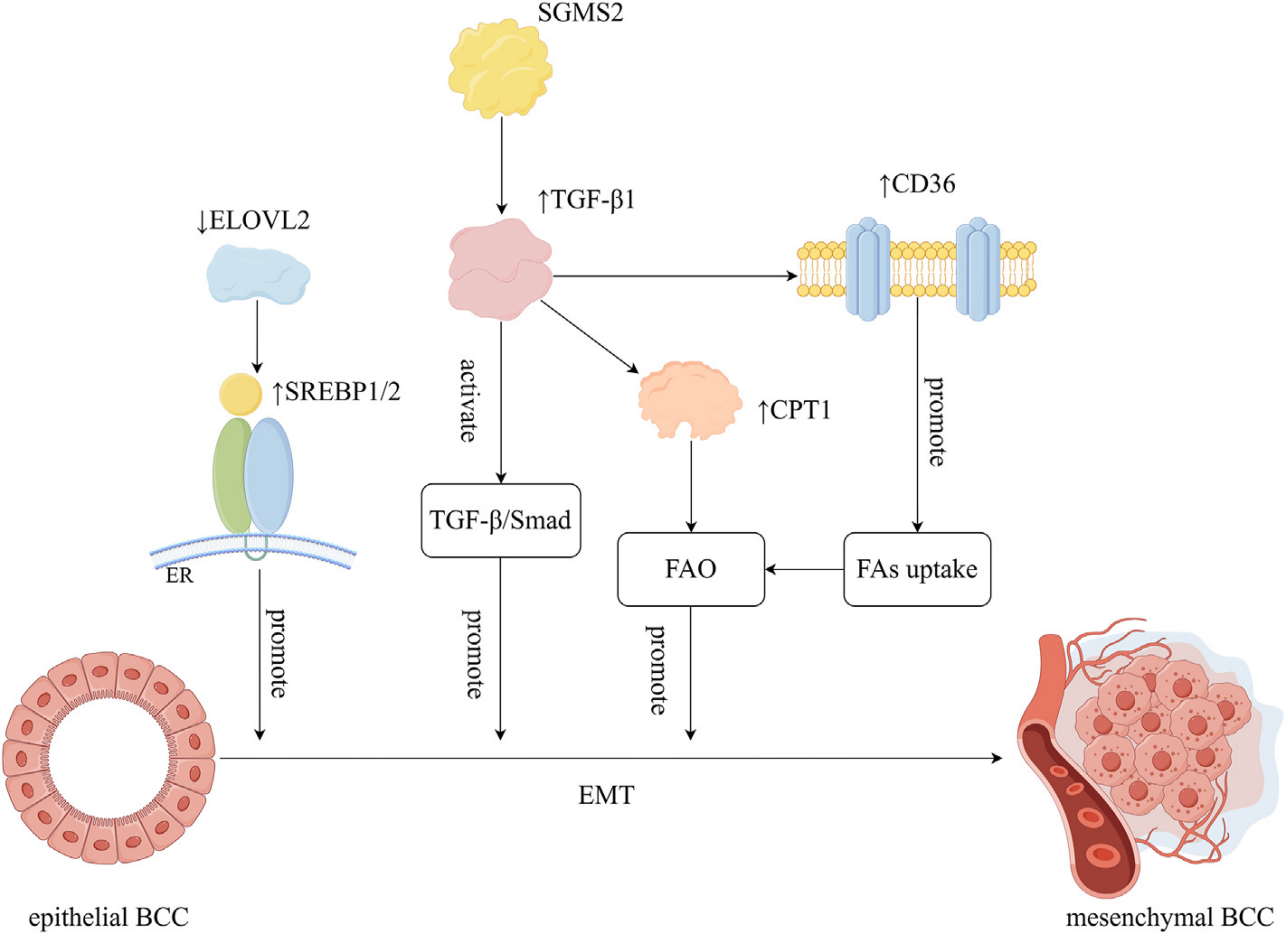
Dysregulation of lipid metabolism promotes EMT (by Figdraw). Down-regulation of ELOVL2 promotes EMT in breast cancer by up-regulating expression of SREBP1/2. SGMS2 promotes EMT by increasing TGF-β1 secretion to activate the TGF-β/Smad signaling pathway; TGF-β1 promotes the expression of CD36 and CPT1, leading to increased FA uptake and FAO, which in turn promotes EMT in BCCs. BCC, breast cancer cell; CD36, cluster of differentiation 36; CPT1, carnitine palmitoyltransferase 1; EMT, epithelial–mesenchymal transition; ELOVL2, elongation of very long chain fatty acids-like 2; FA, fatty acid; FAO, fatty acid oxidation; SGMS2, sphingomyelin synthetase 2; SREBP1/2, sterol regulatory element binding protein 1/2; TGF-β1, transforming growth factor beta 1.

Compared with mesenchymal cells, epithelial cells are characterized by a higher level of monounsaturated FA and higher expression of FASN.^64^ Numerous studies have shown that the lipid composition of BCC changes globally during EMT. Elongation of very long chain fatty acids-like 2 (ELOVL2) is a lipid metabolism regulator. Down-regulation of ELOVL2 can induce lipid metabolism reprogramming and promote EMT in breast cancer by up-regulating the expression of SREBP1/2.^65^ SGMS2, a key regulator involved in Cer and sphingolipid homeostasis, promotes EMT mainly by increasing TGF-β1 (transforming growth factor beta 1) secretion to activate the TGF-β/Smad signaling pathway, thereby enhancing cancer cell invasiveness.^66^ The inhibition of the adipogenic process catalyzed by FASN and the up-regulation of FA uptake used for FAO to produce ATP are both important components of TGF-β1-induced EMT and metastasis in breast cancer^67^ (Fig. 1). The discovery of these dysregulated metabolic pathways provides important ideas for inhibiting the progression of breast cancer.

As mentioned in the sterol metabolism section, SREBP1 is significantly associated with EMT in BBCs.^36^ Alkaline-extracted mycelial polysaccharides inhibit lipid metabolism and EMT in breast cancer through miR-215-5p/SREBP1 axis, showing anti-tumor potential.^68^

**Figure 2:**
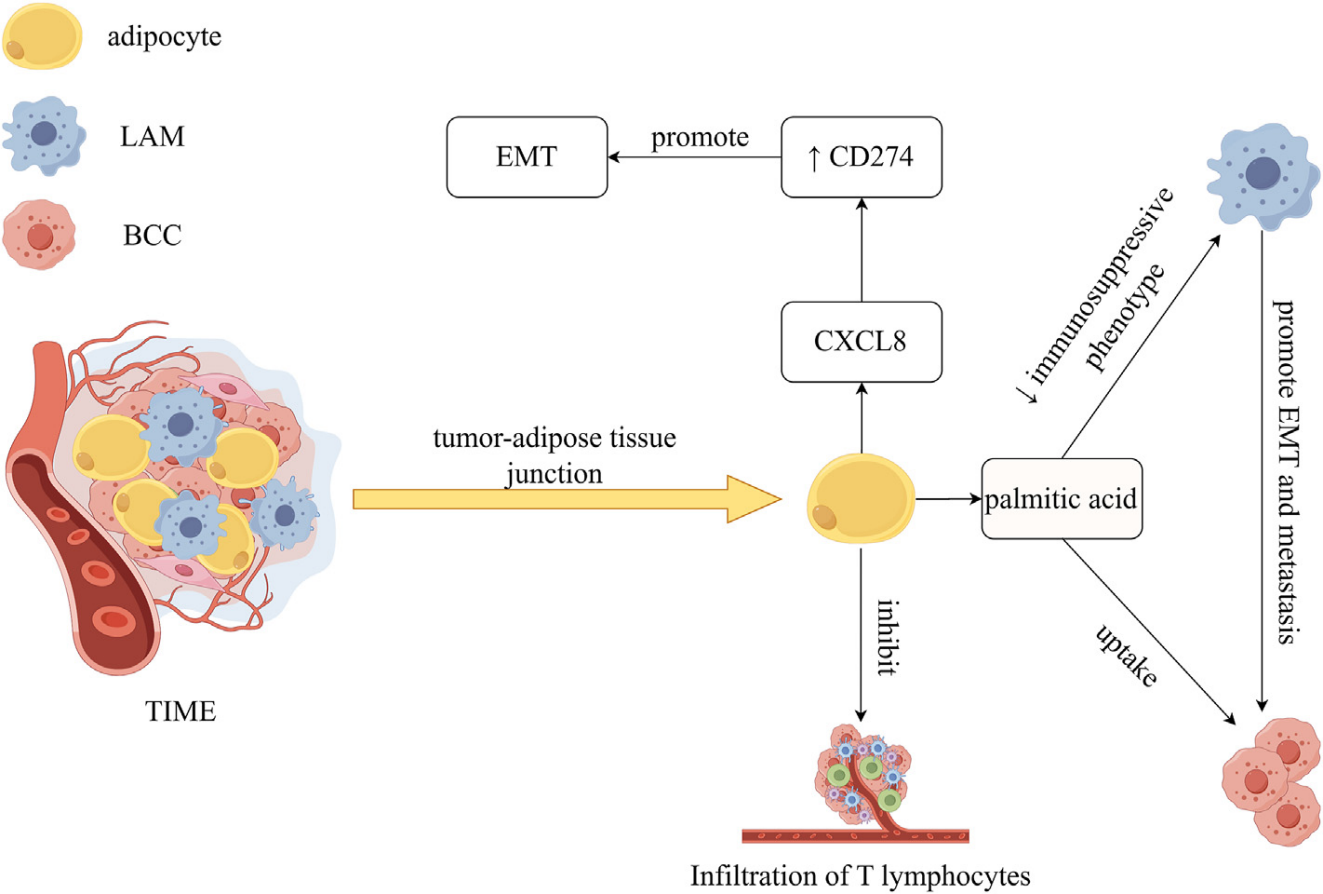
TIME associated with lipids in breast cancer (by Figdraw). Tumor-associated adipocytes secrete palmitic acid; BCCs uptake palmitic acid; LAM is mainly distributed in the tumor-fat junction; M2 macrophages promote EMT and migration of BCCs; palmitic acid reduces the immunosuppressive phenotype of M2 macrophages. CXCL8 is highly expressed in tumor-associated adipocytes; CXCL8 promotes the EMT process of TNBC through the PI3K/AKT pathway. Tumor-associated adipocytes inhibit infiltration of T lymphocytes in breast cancer. AKT, protein kinase B; BCC, breast cancer cell; CD274, cluster of differentiation 274; CXCL8, C–X–C motif chemokine ligand 8; EMT, epithelial–mesenchymal transition; LAM, lipid-associated macrophages; PI3K, phosphoinositide 3 kinase; TIME, tumor immune microenvironment.

Cancer-associated adipocytes, a type of tumor-cultured adipocytes in the TME of TNBC, interact with breast cancer and can secrete various cytokines, adipokines, and chemokines, which lead to changes in cancer cell phenotype and function.^69^^,^^70^ C–X–C motif chemokine ligand 8 (CXCL8) is highly expressed in tumor-associated adipocytes and promotes EMT of TNBC through the PI3K/AKT pathway, thereby promoting the growth and metastasis of TNBC^71^ (Fig. 2).

Lipids, including FAs, cholesterols, and sphingolipids, play a crucial role in promoting the EMT process in BCCs. Further investigation into the association between lipid metabolism and EMT is warranted to advance our understanding of BCC metastasis and to facilitate the development of novel therapeutic strategies for inhibiting BCC metastasis in the future.

## Tumor immune microenvironment (TIME) associated with lipids

In the process of tumor development, macrophages, dendritic cells, neutrophils, B cells, T cells, CAF, and other cells are recruited to the microenvironment around tumor cells, and constitute TIME together with the vasculature and extracellular matrix, cytokines, chemokines, metabolites, and exosomes.^72^ The metabolism of cells in TIME is closely related to the effect of cancer treatment.^73^ The malignant potential of tumor cells, cell culture dimension (2D and 3D), and TIME can significantly affect the lipid biosynthesis of tumor cells.^74^ Here, we introduce the correlation between the lipid metabolism of M2 macrophages, CAF, and CD8^+^ T cells and the progression of breast cancer.

Lipid-associated macrophages in the tumor-adipose microenvironment show high expression levels of genes involved in lipid metabolism (FABP3–5, LPL (lipoprotein lipase), and LIPA (lipase A)) and some lipid receptors (LGALS3 (lectin, galactoside-binding, soluble 1) and TREM2 (triggering receptor expressed on myeloid cells 2)).^75^ Lipid-associated macrophages are characterized by typical functional characteristics of M2-like macrophages and enhanced lipid accumulation and phagocytosis and are mainly distributed in the tumor-fat junction and promote the progression of breast cancer.^75^ Infiltration of M2-like macrophages in the tumor-adipose microenvironment is associated with poor survival in breast cancer patients and elimination of lipid-associated macrophages in the tumor-adipose microenvironment enhances the effect of anti-PD-1 therapy.^75^ M2 macrophages promote EMT and migration of BCCs, and the immunosuppressive phenotype of M2 macrophages can be reduced by palmitic acid.^76^ Interestingly, mature adipocytes can export palmitic acid to BCCs, suggesting a contribution of peri-breast adipocytes to breast cancer progression.^77^ However, exogenous palmitic acid and Cer exert an inhibitory effect on breast cancer progression by simultaneously targeting cancer cells and M2 macrophages^76^ (Fig. 2).

In breast cancer, CD10 is mainly expressed in CAF. CD10 is not only a cell surface marker but also a functional driver of anti-tumor peptide hydrolysis causing stemness and chemotherapy resistance in tumor cells.^78^ Lipids are increased in cancer cells exposed to CAF mediators, and CAF acts as a hub for lipids to support the metabolic demands of BCCs and disease progression.^17^ In obesity-related breast cancer, leptin and PD-1 can inhibit glycolysis in CD8-positive T cells by increasing FAO, thereby promoting breast tumorigenesis.^79^ In TNBC, CXCL8 derived from tumor-associated adipocytes regulates the immunosuppressive microenvironment and promotes tumor progression by up-regulating the expression of CD274 and inhibiting T cell infiltration.^71^

Abnormal lipid metabolism of BCCs and immune cells in the tumor microenvironment, along with their crosstalk, creates a favorable TIME for BCC growth infiltration, metastasis, and drug resistance. Alterations in macrophages, CAF, and T cells are particularly associated with aberrant lipid metabolism. Due to the complexity of TIME components, future researchers need to not only delve into how aberrant lipid metabolism contributes to TIME remodeling but also identify key regulatory hubs to provide new ideas for inducing a tumor-suppressive microenvironment and treating breast cancer.

## Drug resistance related to lipid metabolism

Breast cancer is treated with a variety of treatment options in clinical practice, including surgery, radiotherapy, and chemotherapy. More specific treatments include hormone (endocrine) therapy, targeted therapy, and immunotherapy. Despite numerous treatment modalities, treatment failure and disease recurrence due to drug resistance are still common in all types of breast cancer.

Lipid metabolism disorder plays an important role in the process of drug resistance in breast cancer. In particular, the expression levels of lipid metabolism-related genes in BCC are significantly different between chemosensitive and chemoresistant cells. Here we briefly review the correlation between drug resistance and lipid metabolism in breast cancer.

## Resistance to chemotherapy drugs

Chemotherapy is indicated for invasive breast cancer with lymph node metastasis. Commonly used chemotherapy drugs include antibiotics (doxorubicin, epirubicin, adriamycin, *etc*.), alkylating agents (cyclophosphamide, *etc*.), alkaloids (docetaxel, paclitaxel, vinorelbine, *etc*.), platinum agents (carboplatin, cisplatin, *etc*.), and antimetabolites (fluorouracil, capecitabine, *etc*.).

Adipocytes in mammary TME promote chemoresistance. Adipocyte-secreted factors induce adriamycin resistance by increasing the utilization of inflammatory mediators and inhibiting the release of free fatty acids from adriamycin-treated cells.^80^ Carnitine palmitoyltransferase 1C (CPT1C) is involved in the regulation of lipid metabolism and important liposome changes. CPT1C silencing drives serosa remodeling, leading to chemotherapy resistance to doxorubicin in BCCs, and the low expression of CPT1C is a predictive marker for poor prognosis in HER2-positive breast cancer and TNBC patients treated with anthracycline-based neoadjuvant chemotherapy.^81^

In BCCs, the long-chain free fatty acid receptor GPR120 promotes the *de novo* synthesis of fatty acids that act as potential GPR120 ligands to activate GPR120 signaling through a feedback mechanism.^82^ Up-regulated GPR120 signaling renders BCCs resistant to epirubicin-induced cell death by up-regulating the expression of ATP-binding cassette transporters, thereby reducing intracellular epirubicin accumulation.^82^

FAO up-regulates acyl-CoA synthetase long chain family member 4 (ACSL4) to increase phospholipid biogenesis by increasing STAT3 (signal transducer and activator of transcription 3) acetylation in chemotherapy-resistant TNBC cells.^83^ The increase of phospholipids enhances the mitochondrial membrane potential, which contributes to resist paclitaxel-induced apoptosis.^83^

Cisplatin is a commonly used drug in the chemotherapy of TNBC patients. Studies have shown that valproic acid antagonizes cisplatin resistance by inhibiting FAO, and valproic acid combined with cisplatin can enhance the cancer cell killing ability of cisplatin.^84^

Dysregulated lipid metabolism promotes BCC resistance by changing the composition of the cell plasma membrane, reducing the accumulation of drugs in BCCs, and changing the potential, which indicates that resistant BCCs are very cunning. For BCCs with different drug tolerance, it may be necessary to combine drugs to correct their characteristic metabolic changes to restore the killing effect of drugs.

## Drug resistance in endocrine therapy

Endocrine therapy is a systemic treatment that acts on cancer cells anywhere in the body and is used clinically for the treatment of ER^+^ and/or PR^+^ breast cancer. The commonly used drugs in endocrine therapy for breast cancer include estrogen analogs (tamoxifen, toremifene), ER degraders (fulvestrant), and aromatase inhibitors (letrozole, anastrozole, exemestane). Tamoxifen, which is structurally similar to estrogen, competes with estrogen for ER in target organs and affects gene transcription, thereby inhibiting tumor cell growth and migration.^85^ Since tamoxifen-based endocrine therapy is the mainstay of adjuvant therapy for ER^+^ breast cancer, a major concern with endocrine therapy is the development of acquired resistance in approximately 40% of patients who receive tamoxifen.^86^ Here we only introduce the correlation between tamoxifen resistance and lipid metabolism.

In 2012, the tumor protein MUC-1 was found to up-regulate the transcription of genes encoding cholesterol and lipid metabolism enzymes and was associated with tamoxifen resistance in breast cancer.^87^

Mitochondrial energy metabolism is specifically related to tamoxifen resistance in breast cancer,^88^ in which FAO plays an important role. CPT1 is the rate-limiting enzyme in the FAO process.^89^ FAO activation mediated by activated CPT1 is a key driver of tamoxifen resistance in ER^+^ BCCs.^90^ Both the CPT1 inhibitor etomoxil and the JNK (c-Jun N-terminal kinase) inhibitor blocking the phosphorylation of c-Jun can restore the sensitivity of drug-resistant cells to tamoxifen.^90^ If mitochondrial energy metabolism inhibitors can be developed in combination with endocrine therapy, the problem of resistance to endocrine therapy for breast cancer may be solved.

## Resistance to immune checkpoint inhibitors

Immunotherapy has become a new option for many refractory solid tumors. Immune checkpoint inhibitors (ICI) protect T cells from immunosuppression by cancer cells by blocking immune checkpoints, thereby restoring their anti-tumor activity.^91^ Immune checkpoint inhibitors include inhibitors of programmed death protein 1 (PD-1) and its ligand 1 (PD-L1) and cytotoxic T-lymphocyte-associated antigen 4 (CTLA-4). Although a large number of TNBC patients have a durable response to immune checkpoint inhibitors, unfortunately, many patients who respond initially will later develop acquired resistance.^92^

It has been shown that the expression of LPIN1 is significantly elevated in breast cancer tissues compared with normal breast tissues, and lipid synthesis regulated by LPIN1 promotes BCC migration.^31^ Up-regulation of LPIN1 in breast cancer tissues was inversely correlated with tumor histological grade and p53 activity.^31^ After treatment of breast cancer MCF-7 cells with lithocholic acid, the expression of SREBP1 and FASN decreased, the expression of pro-apoptotic p53 protein increased, and the expression of anti-apoptotic Bcl-2 protein decreased.^93^ A recent study showed that restoring p53 activity sensitizes TNBC to anti-PD-1 therapy.^94^

There are few studies on the correlation between immune checkpoint inhibitor resistance and lipid metabolism, but all seem to point to p53-related alterations. The specific mechanisms by which aberrant lipid metabolism leads to altered p53 expression or activity deserve in-depth investigation.

## Resistance to HER2-targeted therapy

Her2-targeted drugs commonly used in the treatment of breast cancer include trastuzumab (herceptin), patozumab, lapatinib, everolimus, *etc*. Herceptin and lapatinib resistance are described here.

Herceptin has a good therapeutic effect on breast cancer patients with HER2 overexpression, which can reduce the risk of postoperative recurrence and improve the disease-free survival of breast cancer patients.^95^ Bioinformatics analysis revealed that herceptine-resistant cell models exhibited modulation of proteins related to lipid metabolism, organophosphorus biosynthesis, and macromolecular methylation.^96^ CD36 expression is up-regulated in breast cancer patients treated with anti-HER2 therapy (lapatinib, trastuzumab) and is associated with poor prognosis.^12^ FASN may lead to herceptin resistance in breast cancer through at least two pathways: i) up-regulating PEA3 transcription to inhibit HER2 gene expression, and ii) changing the lipid composition and function of BCC membrane, thereby affecting the membrane localization of HER2.^97^

BCCs are more likely to rely on exogenous uptake of FAs in the process of acquiring resistance to HER2 inhibitory therapy, and CD36 is the key up-regulated gene in cells with acquired resistance to HER2 inhibitor lapatinib.^98^ Gene silencing or pharmacological inhibition of CD36 can inhibit the growth of lapatinib-resistant cells, but not lapatinib-sensitive cells.^98^ Clinical data suggest that increased CD36 expression after anti-HER2 therapy is associated with poor prognosis in breast cancer patients.^12^

In summary, in the process of acquiring resistance to HER2 inhibition, CD36 up-regulation allows more FAs to enter the cell for FAO, while FASN up-regulation increases FA biosynthesis and may affect HER2 membrane localization. These alterations are involved in metabolic remodeling and resistance to anti-HER2 therapy. Whether the changes in FA metabolism promote the resistance of BCCs is related to FAO is not clear, and further studies are needed to provide new ideas for overcoming the resistance of HER2 targeted therapy.

## Resistance to CDK4/6 inhibitors

Cyclin D kinase 4/6 (CDK4/6) specifically regulates the transition of cells from the G1 to S phase, and CDK4/6 inhibitors block this process and effectively block the proliferation of sensitive cancer cells.^99^ CDK4/6 inhibitors prolonged progression-free and overall survival in patients with advanced hormone receptor (ER, PR)-positive and HER2-negative breast cancer.^100^ However, resistance to CDK4/6 inhibitors is inevitable, with disease progression occurring in more than 70% of patients within 12–36 months.^101^ So far, there is no strong evidence for the correlation between lipid metabolism and CDK4/6 inhibitor resistance.

## Conclusion

**Figure 3:**
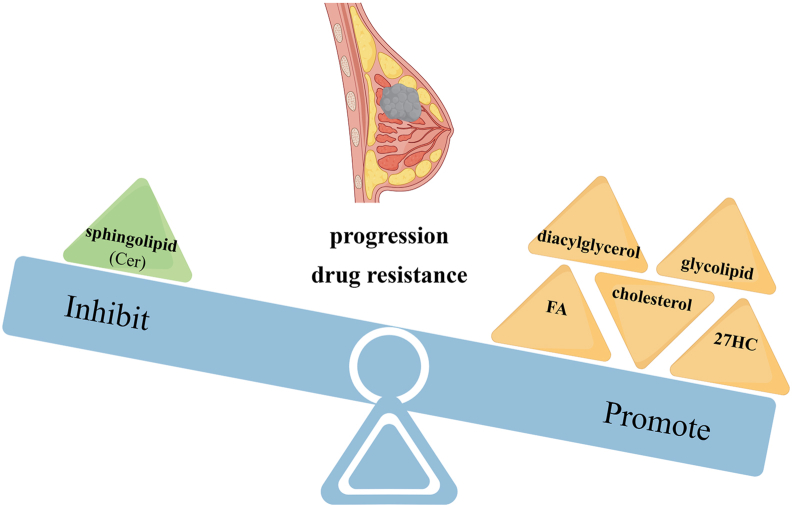
Lipid metabolism involved in progression and drug resistance of breast cancer (by Figdraw). FAs, diacylglycerols, cholesterols and its metabolite 27HC, and glycolipids, promote breast cancer progression and drug resistance development. Ceramides (Cer) inhibit breast cancer progression and drug resistance development. FA, fatty acid; 27HC, 27-hydroxycholesterol.

In summary, the lipid uptake, synthesis, and decomposition of BCCs are significantly altered, especially the metabolism of FAs, cholesterols, sphingolipids, and glycolipids (Fig. 3). The growth, progression, invasion, and drug resistance of BCCs are positively correlated with the increased uptake and biosynthesis of FAs and cholesterols, as well as the up-regulation of FAO. Moreover, BCCs prefer to take FAs from external sources for their development. Cholesterol metabolite 27HC promotes the progression of breast cancer through a variety of ways, and the specific mechanism is very complex. The accumulation of sphingoliths (especially Cer) in cancer cells shows an inhibitory effect on breast cancer. The expression of GD2, a marker of BCSC in TNBC, in breast cancer patients is particularly relevant to the malignant development of breast cancer. Reprogramming of lipid metabolism in breast cancer is a process of changing complex metabolic pathways. Therapies targeting important genes, enzymes, or reaction substrates in lipid metabolism may contribute to the clinical treatment effect of breast cancer.

Although numerous studies have gradually elucidated the association of lipid metabolism with BC progression and drug resistance, many specific mechanisms have yet to be investigated. The study of lipid metabolism and breast cancer progression and drug resistance currently faces numerous challenges. The complexity of the lipid metabolic network makes it difficult to study and requires the integration of multiple bioinformatics and experimental techniques to analyze its effects. Effective ways to apply lipid metabolism findings to clinical breast cancertreatment are lacking, and more clinical trials and translational studies are needed to validate relevant methods and drugs. In addition, due to the differences in breast cancer patient groups, it is difficult to effectively promote and apply personalized treatment based on lipid metabolism research. In the future, the specific mechanisms of lipid metabolism on BBC proliferation, invasion, and drug resistance need to be explored in depth to promote the development of more effective therapeutic approaches.

## Author contributions

**Huijuan Da**i and **Aijun Sun** contributed to the study's conception and design. **Wenxiang Fu** wrote the manuscript. **Wenxiang Fu** and **Huijuan Dai** made the mConflict of interestsanuscript figures.

## Funding

This study was funded by the 10.13039/501100001809National Natural Science Foundation of China (No. 82303865 to Huijuan Dai) and the Science and Technology Commission of Huaian Municipality, Jiangsu, China (No. HAB202209 to Aijun Sun).

## Conflict of interests

The authors declared no competing interests.
